# Features of Invasive Aspergillosis Caused by *Aspergillus flavus*, France, 2012–2018

**DOI:** 10.3201/eid3105.241392

**Published:** 2025-05

**Authors:** Lise Bertin-Biasutto, Olivier Paccoud, Dea Garcia-Hermoso, Blandine Denis, Karine Boukris-Sitbon, Olivier Lortholary, Stéphane Bretagne, Maud Gits-Muselli, Raoul Herbrecht, Valérie Letscher-Bru, François Danion, Sophie Cassaing, Florent Morio, Céline Nourrisson, Marc Pihet, Milène Sasso, Guillaume Desoubeaux, Marie-Fleur Durieux, Julie Bonhomme, Elisabeth Chachaty, Taieb Chouaki, Nicole Desbois-Nogard, Alexandre Alanio, Jean-Pierre Gangneux, Fanny Lanternier

**Affiliations:** Hopital Necker-Enfants Malades, Paris, France (L. Bertin-Biasutto, O. Paccoud, O. Lortholary, F. Lanternier); Université de Paris, Paris (L. Bertin-Biasutto, O. Lortholary, F. Lanternier); Institut Pasteur, Paris (D. Garcia-Hermoso, K. Boukris-Sitbon, O. Lortholary, S. Bretagne, A. Alanio, F. Lanternier); Hôpital Saint Louis, Paris (B. Denis, S. Bretagne, M. Gits-Muselli, A. Aliano); Institut de Cancérologie de Strasbourg, Strasbourg, France (R. Herbrecht); Hôpitaux Universitaires de Strasbourg, Strasbourg (V. Letscher-Bru, F. Danion); Toulouse University Hospital, Toulouse, France (S. Cassaing); Nantes University Hospital, Nantes, France (F. Morio); CHU Clermont-Ferrand, Clermont-Ferrand, France (C. Nourrisson); CHU d'Angers, Angers, France (M. Pihet); CHU Nîmes and Université de Montpellier, Nîmes, France (M. Sasso); Hôpital Bretonneau, Tours, France (G. Desoubeaux); Limoges University, Limoges, France (M.-F. Durieux); Unicaen Université Normandie, Caen, France (J. Bonhomme); Gustave Roussy, Villejuif, France (E. Chachaty); CHU Amiens-Picardie, Amiens, France (T. Chouaki); Inserm U1285, University of Lille, Lille, France (T. Chouaki); Centre Hospitalier Universitaire de Martinique, Fort-de-France, France (N. Desbois-Nogard); Université de Rennes, CHU Rennes, Rennes, France (J.-P. Gangneux); Rennes Teaching Hospital, Rennes (J.-P. Gangneux)

**Keywords:** Aspergillus flavus, fungi, antimicrobial resistance, respiratory infections, aspergillosis, invasive fungal infections, immunosuppression, France

## Abstract

Invasive aspergillosis (IA) caused by *Aspergillus flavus* remains poorly described. We retrospectively analyzed 54 cases of IA caused by *A. flavus* reported in France during 2012–2018. Among cases, underlying IA risk factors were malignancy, solid organ transplantation, and diabetes. Most (87%, 47/54) infections were localized, of which 33 were pleuropulmonary and 13 were ear-nose-throat (ENT) infection sites. Malignancy (70% [23/33]) and solid organ transplantation (21% [7/33]) were the main risk factors in localized pulmonary infections, and diabetes mellitus was associated with localized ENT involvement (61.5%, [8/13]). Fungal co-infections were frequent in pulmonary (36%, 12/33) but not ENT IA (0 cases). Antifungal monotherapy was prescribed in 45/50 (90%) cases, mainly voriconazole (67%, 30/45). All-cause 30-day case-fatality rates were 39.2% and 90-day rates were 47.1%, and rates varied according to risk factor, IA site, and fungal co-infections. Clinicians should remain vigilant for *A. flavus* and consider it in the differential diagnosis for IA.

After invasive candidiasis and pneumocystosis, invasive aspergillosis (IA) is the third most frequent invasive fungal infection in Europe. IA occurs primarily in immunocompromised patients, including those with a hematologic malignancy (HM) and those who receive solid organ transplants or immunosuppressive treatments (*1*). After *A. fumigatus*, *A. flavus* is the second most frequently reported *Aspergillus* species isolated from clinical specimens in invasive and noninvasive aspergillosis, but *A. flavus* IA has marked differences in infection sites and geographic locations. Indeed, *A. flavus* is reported as the main etiologic agent of sinusitis, keratitis, and invasive aspergillosis in the Middle East, northern Africa, and South Asia (*2*–*4*). In stark contrast, *A. flavus* accounts for <10% of IA cases reported in Europe and North America (*5*,*6*). For instance, according to data from the Fungal Infection Surveillance Network (RESSIF; RESeau de Surveillance des Infections Fongiques) in France, *A. flavus* represented 8.7% (74/845) of all IA during 2012–2018, second only to the 86% of IA caused by *A. fumigatus*.

Data regarding the epidemiology, risk factors, clinical manifestations, and management of IA caused by *A. flavus* remain scarce, particularly in Europe. We sought to describe patient characteristics, underlying conditions, clinical presentations, and outcomes of IA cases caused by *A. flavus* reported in France.

## Material and Methods

### Database Management and Case Definition

The RESSIF (*1*,*7*), based at the French National Reference Center for Invasive Mycoses and Antifungals (NRCMA), Institut Pasteur, Paris, is a nationwide surveillance network for cases of invasive mycoses in France. RESSIF relies on the active participation of 21 collaborative centers in 15 of the 18 regions of France, including overseas territories. Case details are sent to the NRCMA by referent medical mycologists in collaboration with clinicians.

We included all patients registered in RESSIF during 2012–2018 with proven or probable IA caused by *A. flavus*, according to the 2020 European Organization for Research and Treatment of Cancer/Mycoses Study Group Education and Research Consortium criteria (*8*), which include diabetes mellitus and severe burns as additional risk factors for probable IA. We excluded cases for which data were unavailable and cases of noninvasive aspergillosis (e.g., aspergilloma). We considered the date of IA diagnosis to be the date of the first microbiological criteria leading to diagnosis.

### Data Collection and Classification

Data were collected by using a standardized case report form that included patient demographic characteristics, clinical and radiologic manifestations, time from symptom onset to diagnosis, diagnostic methods, treatment regimen, and 30- and 90-day case-fatality rates (CFRs). We assigned each patient to 1 of 6 main underlying condition categories: HM, including acute leukemia (myeloid or lymphoid) and other lymphoid malignancies; solid organ tumors; solid organ transplant; diabetes mellitus; severe burns; other risk factors, such as HIV and iatrogenic agranulocytosis; or no risk factors, in the absence of all other conditions. For patients with multiple potential risk factors, we prioritized HM and solid organ transplantation. We considered patients with HM, solid organ tumors under chemotherapy treatment, solid organ transplants, and other risk factors to be severely immunocompromised and grouped them for statistical comparisons; we did not include patients with severe burns in that group because, although immunocompromised, those case-patients have specific features. We considered patients with diabetes moderately immunocompromised and considered patients without risk factors to be immunocompetent.

We defined dissemination as infection in >2 noncontiguous sites. We separated localized IA into pleuropulmonary, ear-nose-throat (ENT), central nervous system (CNS), and skin disease. We considered cases with ENT and skull base infection sites as localized ENT infections with contiguous skull base extension. We considered cases with pleuropulmonary and ENT sites of infection as localized pulmonary infections with a contiguous sinus localization. We defined neutropenia as a neutrophil polymorphonuclear cell count of <0.5 × 10^6^ cells/mL. We considered positive galactomannan (GM) antigen in blood samples only, with a positivity threshold of >0.5 optical density. We defined bacterial, viral, or fungal co-infections as microbial pathogens found in the same microbiological samples as *A. flavus* at time of diagnosis and compatible with the patient’s clinical manifestations.

Because *A. flavus* has higher prevalence in dry and warm regions (*2*–*4*), we evaluated seasonality of infections in France. We chose April 1–October 31 as the warmest months of the year in France, with average temperatures of >14°C, compared with November 1–March 31, during which average temperatures were <10°C, according to the 2018 report from the national meteorological organization in France (*9*).

### Species Identification and Antifungal Susceptibility Testing

NRCMA centralized all isolates for identification confirmation. NMRCA identified *A. flavus* by morphology and partial DNA sequence analysis of the calmodulin gene. We only retained *A. flavus* var. *flavus*. We performed in vitro susceptibility testing according to the European Committee on Antimicrobial Susceptibility Testing (EUCAST) broth microdilution method (https://www.eucast.org/fileadmin/src/media/PDFs/EUCAST_files/AFST/Files/EUCAST_EDef_9.4_method_for_susceptibility_testing_of_moulds.pdf) with some modifications. NRCMA determined MICs for 7 antifungal agents, including liposomal amphotericin B (L-AmB), isavuconazole, voriconazole, posaconazole, itraconazole, caspofungin, and micafungin.

### Statistical Analyses

We calculated continuous variables by using Mann-Whitney nonparametric test and expressed results as medians and interquartile range (IQRs). We used Fisher exact test for categorical variables and expressed results as frequencies and percentages. We performed all analyses in Stata 17 (StataCorp LLC, https://www.stata.com) and considered p<0.05 to be statistically significant.

### Ethics

This research was performed in compliance with French law and the Declaration of Helsinki as adopted in 2000. NRCMA monitoring was approved by the Institute Pasteur institutional review board 1 (approval no. 2009–34 IRB) and the Commission Nationale de l’Informatique et des Libertés (National Commission for Information Technology and Civil Liberties) according to regulations in France.

## Results

### Patient Characteristics and Underlying Conditions

During January 2012–December 2018, a total of 54 patients with proven (44.4%) or probable (55.6%) IA caused by *A. flavus* were reported to RESSIF. Median patient age was 58 (IQR 48–70) years; 48.1% were male and 51.8% were female (Table 1). The main underlying condition was HM (53.7%, 29/54), most frequently acute leukemia (20/54, including 10 with acute myeloid leukemia and 10 with acute lymphoid leukemia) (Table 1). Almost all (90%, 26/29) HM patients had neutropenia at diagnosis. The second most frequent underlying condition was solid organ transplant (16.7%, 9/54), including kidney (n = 3), heart (n = 3), lung (n = 2), and liver (n = 1) transplantation. Median time between transplantation and IA was 5 months, with differences according to the type of organ transplant, from 1 month for heart transplant recipients to 36 months for kidney transplant recipients. Eight (14.8%) patients had diabetes mellitus as the sole risk factor. The other patients had solid organ tumors under chemotherapy (n = 2), severe burns (n = 2), HIV infection (n = 1), or carbimazole-induced agranulocytosis (n = 1); 2 patients had no identified risk factors.

**Table 1:** Characteristics of 54 patients with probable or proven invasive aspergillosis caused by *Aspergillus flavus*, France, 2012–2018*

Characteristics	Value
Median age, y (IQR)	58 (48–70)
Originating from Africa, n = 50	14 (28)
Diagnosis during April 1­–Oct 31	32 (59.2)
Sex	
M	26 (48.1)
F	28 (51.8)
Primary underlying risk factor	
Malignancy	31 (57.4)
Solid organ cancer	2 (3.7)
Hematologic malignancy, n = 29	29 (54)
Acute myeloid leukemia	10 (34.5)
Acute lymphoid leukemia	10 (34.5)
Lymphoma	2 (6.9)
Chronic lymphoid leukemia	2 (6.9)
Other hematologic malignancy	5 (17.2)
Solid organ transplant, n = 9	9 (16.7)
Kidney	3 (33.3)
Heart	3 (33.3)
Lung	2 (22.2)
Liver	1 (11.1)
Diabetes mellitus	8 (14.8)
Severe burns	2 (3.7)
Other immunodeficiency, n = 2	2 (3.7)
HIV	1 (50)
Iatrogenic agranulocytosis	1 (50)
No risk factor	2 (3.7)
Median time between onset of symptoms and diagnosis, d (IQR)	20 (10–50)
Site of infection	
Localized infections, n = 47	47 (87)
Pulmonary†	33 (70.2)
Ear-nose-throat†	13 (27.7)
Cerebral contiguous extension‡	10 (21.3)
Skin and soft tissue	1 (2.1)
Disseminated infections, n = 7	7 (13)
Pulmonary	6 (85.7)
Ear-nose-throat	2 (28.8)
Cerebral	2 (28.8)
Skin and soft tissue	5 (71.4)
Fungemia	2 (28.6)
Positive serum galactomannan antigen, n = 38	23 (60.5)
Proven infection	24 (44.4)
Fungal coinfection at same site as *A. flavus*, n = 16§	16 (29.7)
* A. fumigatus*	6 (37.5)
* A. niger*	3 (18.8)
Mucorales	5 (31.2)
* Pneumocystis jirovecii*	3 (18.8)
Candidemia	1 (6.2)
Influenza coinfection	2 (3.7)
First-line antifungal therapy, including combinations, n = 50	
Voriconazole	32 (64)
Posaconazole	3 (6)
Itraconazole	2 (4)
Isavuconazole	2 (4)
Liposomal amphotericin B	9 (18)
Echinocandin	7 (14)
Antifungal combination	5 (10)
Death, n = 51 patients with outcome data	
30-day case-fatality, all causes	20 (39.2)
90-day case-fatality, all causes	24 (47.1)

*Values are no. (%) patients except as indicated. †Four patients had lung and ear-nose-throat infections. ‡All were sinus infections with contiguous cerebral lesions. §Including 1 case with 3 fungal co-infections.

Patients with diabetes were significantly older (median age 72.5 [IQR 70–74] years) than patients with malignancy, either HM or solid organ cancer (median age 54 [IQR 32–66] years), or solid organ transplantation (median age 60 [IQR 53.5–70.5] years) (p<0.001) (Table 2). Patients with diabetes also had longer time between symptom onset and diagnosis (median 105 [IQR 68–163] days) than HM patients (median 16 [IQR 5–30] days) or patients with solid organ transplant (median 20 [IQR 13.5–54] days). Pleuropulmonary involvement was more frequent in patients with HM, solid organ tumors, or solid organ transplant (88%, 30/34) than those with diabetes (0/8) (p<0.001). In contrast, all 8 patients with diabetes mellitus had ENT infections, and most (88%, 7/8) had extension to the skull bases, but ENT infections were much less frequent (12%, 4/34) among HM, solid organ tumor, and solid organ transplant patients (p<0.001). We noted no cases of disseminated IA in patients with diabetes, compared with 5 cases (16%, 5/31) in HM patients and 1 (11%, 1/16) case in a solid organ transplant patient (p = 0.57).

**Table 2:** Main underlying risk factors for 54 patients with probable or proven invasive aspergillosis caused by *Aspergillus flavus*, France, 2012–2018*

Characteristics	HM, n = 29	SOC, n = 2	SOT, n = 9	DM, n = 8	Other, n = 2	Severe burns, n = 2	No risk factors, n = 2	p value†
Median age, y (IQR)	53 (28–66)	73 (66–80)	60 (57–70)	72.5 (70–74)	49 (48–50)	60 (50–69)	21 (16–25)	**<0.001**
Originating from Africa, n = 26	5/26 (19.2)	0	2/8 (25)	5 (62.5)	0	0	2 (100)	**0.02**
Sex								
M	12 (41.4)	1 (50)	5 (55.6)	5 (62.5)	1 (50)	0	1 (50)	0.46
F	17 (58.6)	1 (50)	4 (44.4)	3 (37.5)	1 (50)	2 (100)	1 (50)	0.46
Median delay to diagnosis, d (IQR)	17 (5–30)	5 (2–7)	20 (13.5–54)	105 (68–163)	32 (20–43)	7 (5–9)	­NA	**<0.001**
Neutropenia‡§	26 (89.6)	0	1/8 (12)	0	1 (50)	0	0	**<0.001**
Positive serum galactomannan antigen§	15/22 (68.1)	1/1 (100)	3/7 (43)	2/4 (50)	1 (50)	1 (50)	0	0.63
Infection site								
Disseminated infection	4 (13.7)	1 (50)	1 (11.1)	0	0	1 (50)	0	0.57
Localized infection	25 (86.2)	1 (50)	8 (88.9)	8 (100)	2 (100)	1 (50)	2 (100)	0.57
Pleuropulmonary	23/25 (92)	0	7/8 (87.5)	0	2 (100)	0	1 (50)	**<0.001**
ENT	2/25 (8)	1/1 (100)	1/8 (12.5)	8/8 (100)	0	0	1 (50)	**<0.001**
CNS	0	1/1 (100)	1/8 (12.5)	7/8 (87.5)	0	0	1 (50)	**<0.001**
Skin and soft tissue	0	0	0	0	0	1 (100)	0	>0.999
First-line antifungal therapy¶	N = 27		N = 7					
Voriconazole	18 (66.7)	2 (100)	3 (4.8)	6/8 (75)	1 (50)	1 (50)	1 (50)	0.69
L-AmB	5 (18.5)	0	2 (28.6)	0/8 (0)	1 (50)	1 (50)	0	0.32
Other agent	6 (22.2)	0	2 (28.6)	3/8 (37.5)	1 (50)	0	1 (50)	0.41
Death	N = 28	N = 1	N = 8					
30-d CFR	13 (46.4)	0	4 (44.4)	0	1 (50)	2 (100)	0	**0.017**
90-d CFR	15 (53.6)	0	5 (55.6)	1 (12.5)	1 (50)	2 (100)	0	0.052

*Values are no. (%) except as indicated. Bold font indicates statistical significance. CFR, case-fatality rate; CNS, central nervous system; DM, diabetes mellitis; ENT, ear-nose-throat; HM, hemolytic malignancy; IQR, interquartile range; L-AmB, liposomal amphotericin B; NA, not applicable; SOC, solid organ cancer; SOT, solid organ transplant. †For solid organ transplant, solid organ cancer, hemolytic malignancy, and other versus diabetes mellitis. ‡<0.5 × 10^3^ cells/mL §No. positive/no. tested (%). ¶Including combination therapy. Other first-line agents included posaconazole, itraconazole, isavuconazole, and echinocandin.

### Infection Sites and Clinical Manifestations

Of the 54 cases, 47 (87%) were localized (Table 3) and 7 (12.3%) disseminated (Table 4). Among the 47 localized infections, 33 were (70.2%) pleuropulmonary infections and 13 (27.6%) were ENT infections; 10 (77%) of the ENT infections had extension to the skull bases. No isolated CNS infections and only 1 case of localized skin and soft tissue infection were reported.

**Table 3:** Characteristics of localized invasive aspergillosis cases caused by *Aspergillus flavus*, France, 2012–2018*

Characteristics	Localized pleuropulmonary infection, n = 33	Localized ENT infection, n = 13	p value
Median age, y (IQR)	57 (48–67)	72 (61–74)	**0.03**
Originating from Africa	6/29 (21)	7/13 (54)	0.068
Sex			
M	16 (48.5)	7 (53.8)	>0.999
F	17 (51.5)	6 (46.2)	>0.999
Primary risk factors			**<0.001**
Hemolytic malignancy	23 (69.7)	2 (15.4)	**0.001**
Solid organ cancer	0	1 (7.7)	0.28
Solid organ transplant	7 (21.2)	1 (7.7)	0.41
Diabetes mellitus	0	8 (61.5)	**<0.001**
Other	2 (6.1)	0	>0.999
No risk factors	1 (3)	1 (7.7)	0.49
Clinical signs and symptoms			
Fever	22 (66.7)	5 (38.5)	0.1
Dyspnea	18 (54.5)	0	**<0.001**
Cough	11 (33.3)	0	**0.02**
Hemoptysis	4 (12.1)	0	0.09
Otalgia, otorrhea	0	8 (61.5)	**<0.001**
Chemosis, exophthalmia	2 (6.1)	2 (15.4)	0.56
Facial nerve palsy, headache	2 (6.1)	3 (23.1)	0.13
No symptoms	1 (3)	0	>0.999
Imaging features			
Nodules, mass lesion	17 (51.5)	0	0.11
Alveolar consolidation, ground-glass opacities	32 (96.9)	0	**<0.001**
Pleural effusion	18 (54.5)	0	0.11
Sinus opacification	4 (12.1)	12 (92.3)	**<0.001**
Sinus or mastoids lytic lesions	2 (6.1)	9 (69.2)	**<0.001**
Median time to diagnosis, d (IQR)	19 (12–30)	90 (42–163)	**<0.001**
Positive serum galactomannan antigen†	14/26 (53.8)	4/7 (57.1)	1
Diagnostic method‡			>0.999
Smear sputum	9/10 (90)	0	**<0.001**
BAL direct examination	15/25 (60)	NA	NA
BAL culture	24/25 (96)	NA	NA
Biopsy direct examination	5/7 (71)	10 (76.9)	>0.999
Biopsy culture	6/7 (86)	13 (100)	0.35
Proven cases	6 (18)	13 (100)	**<0.001**
Associated localizations			
Pulmonary	NA	0	NA
Ear, nose, throat	4 (12.1)	NA	NA
Central nervous system	0	10 (76.9)	**<0.001**
Mediastinal	1 (3)	0	**<0.001**
Fungal coinfection at same site§	12 (36.3)	0	**0.01**
* Aspergillus fumigatus*	5(15.1)	0	0.31
* A. niger*	3 (9.1)	0	0.55
Mucorales	4 (12.1)	0	0.31
* Pneumocystis jirovecii*	2 (6.1)	0	>0.999
Influenza co-infection	2 (6.1)	0	>0.999
First-line antifungal monotherapy¶	26/30 (86.7)	11/12 (91.7)	>0.999
Voriconazole	16/26 (61.5)	8/11 (72.7)	0.71
Other triazole	4/26 (15.3)	3/11 (27.3)	0.40
Echinocandin	2/26 (7.7)	0/11	>0.999
Liposomal amphotericin B	4/26 (15.4)	0/11	0.30
Antifungal combination therapy¶	4/30 (13.3)	1/12 (8.3)	>0.999
Curative surgery	3 (9.1)	6 (46.1)	**0.01**
Death	N = 31	N = 12	
30-day case-fatality	14 (45.2)	1 (8.3)	**0.03**
3-month case-fatality	17 (54.8)	2 (16.7)	**0.04**

*Values are no. (%) except as indicated. BAL, bronchoalveolar lavage; IQR, interquartile range. †Positive galactomannan antigen in blood with an optical density index cutoff value of >0.5. ‡Values are no. positive/no. tested (%). §13 cases with pulmonary fungal coinfections, including one with more than 1 coinfection. ¶Values are no. positive/no. treated (%).

**Table 4:** Main characteristics of patients with disseminated infections in a study of features of invasive aspergillosis caused by *Aspergillus flavus*, France, 2012–2018*

Characteristics	Value
Median age, y (IQR)	36 (14.5–58)
Sex	
M	3 (42.8)
F	4 (57.1)
Main risk factor	
Hematologic malignancy	4 (57.1)
Solid organ cancer	1 (14.3)
Solid organ transplant	1 (14.3)
Others, severe burns	1 (14.3)
Fever as primary clinical sign	5 (71.4)
Site of infection	
Pulmonary	6 (85.7)
Ear, nose, throat	2 (28.6)
Central nervous system	2 (28.6)
Skin and soft tissues	3 (42.8)
Fungemia	2 (28.6)
Median time to diagnosis, d (IQR)	5 (4.5–28)
Positive serum galactomannan antigen, n = 5	5 (100)
Proven cases	5 (71.4)
Fungal co-infection	3 (42.8)
Voriconazole first-line antifungal therapy	6 (85.7)
3-month case-fatality	4 (57.1)

*IQR, interquartile range.

Compared with patients with pleuropulmonary IA, those with ENT infections were significantly older (median age 72 [IQR 61–74] years vs. 57 [IQR 48–67] years; p = 0.03) and had a longer duration of symptoms before diagnosis (median 90 [IQR 42–163] days vs. 19 [IQR 12–30] days; p = 0.0005). Patients with pleuropulmonary IA had higher rates of HM and solid organ transplantation than patients with ENT IA (69.7% [23/33]) vs. 15.4% [2/13] for HM, and 21.2% [7/33] vs. 15.4% [1/13] for solid organ transplantation; p<0.001). In contrast, diabetes mellitis was the most common underlying condition in patients with ENT IA (61.5% [8/13]), compared with no diabetes mellitis in patients with pleuropulmonary IA (p<0.001). 

We also assessed available imaging results for the 54 included IA cases. All 33 patients with pleuropulmonary IA had chest computed tomography scans, 17 (51.5%) of whom had nodules, 32 (96.6%) had alveolar consolidations or ground-glass opacities, and 18 (54.5%) had pleural effusion. All 13 ENT IA patients had computed tomography scans of the sinuses, ear, or brain, showing sinus opacification in 12 (92.3%) and lytic lesions of sinus, mastoid, or skull base walls in 9 (69.2%) cases. When performed, serum GM testing was positive in 53.8% (14/26) of cases of pleuropulmonary IA and in 57.1% (4/7) of ENT IA cases.

Among pulmonary IA cases, 36.4% (12/33) of patients had >1 fungal co-infection at the same site; 11 had 1 co-infection and 1 had 3 fungal co-infections. Co-infections included 5 *A. fumigatus*, 3 *A. niger*, 4 Mucorales, and 2 *Pneumocystis jirovecii*. Patients with ENT IA did not exhibit relevant fungal co-infections. Of note, influenza co-infection was reported in 6.1% (2/33) of pulmonary IA. In those cases, influenza was diagnosed before IA, and the diagnosis was considered influenza-associated pulmonary aspergillosis.

Among the 7 disseminated IA cases, the most frequent underlying condition was HM in 4 (57%) cases, followed by 1 (14.3%) case each of solid-organ tumor under chemotherapy, solid organ transplant, and severe burns. Patients with disseminated IA infections were younger (median age 36 [IQR 14–66] years) than patients with localized IA (median age 60 [IQR 50–70] years; p = 0.04). The most frequent sites involved in disseminated IA were pulmonary (85.7%), skin and soft tissues (42.8%), ENT (28.6%), and CNS (28.6%). Serum galactomannan was positive in all disseminated cases. Two patients had *A. flavus* fungemia (1 patient had metastatic cholangiocarcinoma and 1 had severe burns). Fungal co-infections were found in 43% (3/7) of cases, including various pathogens identified in different sites: 1 *P. jirovecii* in a bronchoalveolar lavage sample associated with *A. flavus* cutaneous infection, and 1 candidemia and 1 sinusal mucormycosis associated with *A. flavus* found in a puncture of a frontonasal subcutaneaous collection. Finally, disseminated IA with pulmonary involvement (6/7) accounted for 15.4% (6/39) of all cases of pulmonary IA, and disseminated IA with ENT involvement (2/7) accounted for 13.3% (2/15) of all ENT IA.

### Treatment and Outcomes

Before invasive *A. flavus* infection, only 3 patients, all of whom had HM, had been receiving antifungal prophylaxis (1 fluconazole and 2 L-AmB). An antifungal treatment was recorded in 50 (92.6%) of 54 cases. The other 4 (7.4%) patients did not receive antifungal therapy: 3 patients had localized pleuropulmonary IA cases diagnosed postmortem, and the fourth patient had a complete curative surgical treatment for ENT IA. Antifungal monotherapy was prescribed as the first-line treatment for 45 (90%) of the 50 patients who received antifungal treatment, most (66.7%, 30) of whom received voriconazole, but other azoles (posaconazole in 7%, itraconazole in 4%, and isavuconazole in 4%) were also administered, as were L-AmB in 13% (n = 6) and an echinocandin in 4% (n = 2). Five (10%) patients received combined antifungal therapy, including L-AmB and an echinocandin in 3 cases and voriconazole and an echinocandin in 2 cases. Of the 54 ctotal cases, curative surgery was performed in 9 (16.6%), more frequently in ENT IA (46.1%, 6/13) cases than in pleuropulmonary IA (3%, 1/33) cases (p = 0.01).

We determined EUCAST MICs for itraconazole, posaconazole, voriconazole, isavuconazole, L-AmB, caspofungin, and micafungin on 46 isolates (Table 5). The MICs for amphotericin B ranged from 0.5 to >4 mg/L, and the MICs for voriconazole ranged from 0.125 to 1 mg/L. *A. flavus* showed lower susceptibility to amphotericin B (MIC_50_ of 2 mg/L and MIC_90_ of 4 mg/L) than to voriconazole (MIC_50_ of 0.5 mg/L and MIC_90_ of 1 mg/L).

**Table 5:** MICs of first-line antifungal drugs used for 46 cases of invasive aspergillosis caused by *Aspergillus flavus*, France, 2012–2018*

Antifungal drug	Range, mg/L	MIC_50_	MIC_90_
Voriconazole	0.125–1	0.5	1
Isavuconazole	0.25–2	0.5	1
Itraconazole	0.06–0.5	0.125	0.25
Posaconazole	<0.015–0.25	0.125	0.25
Liposomal amphotericin B	0.5 to >4	1	4
Caspofungin	0.125–0.5	0.25	0.5
Micafungin	0.007–0.03	0.007	0.015

*Values according to European Committee on Antimicrobial Susceptibility Testing broth microdilution method (https://www.eucast.org/fileadmin/src/media/PDFs/EUCAST_files/AFST/Files/EUCAST_EDef_9.4_method_for_susceptibility_testing_of_moulds.pdf). MIC_50_, MIC that inhibited 50% of tested microorganisms; MIC_90_, that inhibited 90% of tested microorganisms.

Among 51 patients with available outcome data, 20 patients died, yielding a 30-day CFR of 39.2% (95% CI 25.8%–53.9%) (Table 1). The CFR was higher (46%) for patients with malignancy or solid organ transplant than for patients with diabetes mellitus (n = 0) (p = 0.013) (Table 2). Although not statistically significant, CFR was higher for disseminated (57%) than localized (36%) infections (p = 0.4). Among the different localized IA, pleuropulmonary IA had a higher CFR than did ENT IA (45% vs. 8%; p = 0.03) (Table 3). IA associated with fungal co-infections tended to have a higher 30-day CFR than those without fungal coinfections (44% [7/16] vs. 37% [13/35]; p = 0.8).

## Discussion

Herein, we describe 54 cases of invasive aspergillosis caused by *A. flavus* reported in France during 2012–2018. Few data on *A. flavus* IA in the Northern Hemisphere are available, probably because *A. flavus* is scarce in high-income countries, cases are underdiagnosed because of nonspecific clinical manifestations and absence of diagnostic confirmation in mildly immunocompromised patients, and most countries do not have specific fungal surveillance programs. Thus, we sought to identify risk factors specific to *A. flavus* and the association of risk factors with clinical manifestations and outcomes. 

Although some characteristics of our patients, such as median age (58 years) and CFR (42%), were similar to cases of IA (all species combined) reported in France during 2005–2007 (*7*) and 2012−2018 (*1*), we found a relatively lower percentage (55%) of patients with HM and a higher percentage (14.8%) with diabetes among the underlying factors, compared with 71% for patients with HM and <10% for those with diabetes mellitus from in an earlier study (*1*). Because *A. flavus* has a higher prevalence in dry and warm regions (*2*–*4*), we also investigated the possibility of *A. flavus* acquisition in tropical or subtropical countries such as northern or southern Africa and East Asia, where patients might have lived. Of note, although recent travel history was not recorded for all patients in our study, 28% of patients originated from Africa, suggesting acquisition of *A. flavus* in the Southern Hemisphere. 

We also assessed seasonality of infections in France. We found a higher rate (59.2%) of diagnoses of IA caused by *A. flavus* in the hottest months (Table 1), highlighting the probable influence of hot temperatures on *A. flavus* development, as previously reported (*10*).

We identified 2 distinct patterns of disease: pulmonary IA in highly immunocompromised patients, and ENT IA primarily occurring in patients with diabetes mellitus. Most (87%) cases of IA in our study were localized pleuropulmonary infections. Characteristics of infection were similar in terms of underlying condition, clinical manifestations, and prognosis to previously reported IA cases caused by *A. fumigatus* (*11*). HM and solid organ transplantation accounted for 91% of underlying risk factors in IA cases, consistent with the >85% reported on pulmonary IA from all species combined in a previous study (*11*). Clinical manifestations of *A. flavus* pulmonary IA among our patient cohort were similar to those for pulmonary IA caused by *A. fumigatus*, including acute symptom onset and a short diagnostic delay. ENT IA was the second main site of infection in our study, which is consistent with prior data reporting *A. flavus* as the main causative species in ENT IA in tropical and subtropical countries (*2**–**4*). ENT infection might be related to the larger size of *A. flavus* conidia compared with that of *A. fumigatus* (*12*,*13*), making progression through the lower airways difficult. 

The main underlying condition among our cohort was uncontrolled diabetes mellitus, which we noted in 61.5% of cases. The association between diabetes mellitus and IA was previously observed in aspergillosis otitis in a study where all 12 patients had diabetes mellitis (*14*) and another in which where 46.2% of patients were diabetic (*15*). The association between diabetes mellitus and IA has been assumed to be the result of susceptibility to functional impairment of innate immunity because of alteration of phagocytosis and efficiency of neutrophil polymorphonuclear cells that are known to play a pivotal role in antifungal immunity (*16*). We noted a higher (54%) percentage of patients originating from North Africa, where environmental prevalence of *A. flavus* is high, among cases of ENT IA compared with 21% among patients with pulmonary IA (p = 0.068). Despite less acute progression of infection in those forms, ENT IA cannot be considered benign, considering the frequent (77%) extension to the skull base reported in our study. That finding is similar to 2 meta-analyses of osteoarticular aspergillosis reported during 1936–2013 (*17*,*18*), wherein skull base osteomyelitis accounted for 18% of cases, 68% of which were related to a contiguous ENT injury.

Concerning antifungal drug therapy, voriconazole, the first-line recommended treatment in current guidelines (*19*), was only used in 64% of cases in this study, similar to data reported in previous epidemiologic studies (*7*). The use of other first-line antifungal drugs, mainly in L-AmB (23%) and echinocandins (20%) in pulmonary IA, could be explained by initial disease severity and diagnostic uncertainty in severely immunocompromised patients with frequent fungal co-infections. Consistent with prior studies (*20*), we found that MICs for L-AmB were high. However, because of the retrospective design of the study, we could not evaluate whether the use of L-AmB as initial therapy was associated with increased mortality rates. Curative surgical management was also prevalent (46%) for ENT IA in our study, consistent with a previous case series of ENT aspergillosis (*15*). Research on surgical therapy, which has been shown to influence illness and death, deserves to be better codified (*21*). Despite rapid initiation of treatment, overall mortality rates remained high (47% at 3 months), with notable variability according to underlying risk factors and IA localizations (17% in ENT vs. 55% in pulmonary infections).

One of the main limitations of our study remains the low number of cases reported, which limits the statistical power of comparisons between groups, needing further collaborative studies among countries in Europe or more broadly to increase the number of cases assessed. Another limitation is a lack of comparison between groups. We compared general characteristics of *A. flavus* IA patients, including sex ratio, age, underlying conditions at diagnosis, and mortality rates, to all cases of IA reported from RESSIF for all *Aspergillus* species, >86% of which were *A. fumigatus*, for the same period (2012–2018) (*1*). However, we could not compare specific subgroups, such as those with localized infections, because of the absence of individual patient data. International studies would also increase the number of cases studied and enable comparisons with infections caused by other non–*A. fumigatus* species. 

*A. flavus* IA could represent an increasing issue in the Northern Hemisphere, and further studies are needed to clarify its prevalence and risk factors. Indeed, during 2005–2007 in France, 3% of reported IA cases were caused by *A. flavus* (*7*), which increased to 8.7% during 2012–2018 (*1*). That increase is probably to the result of multiple reasons, including climate, migration, and rates of immunodeficiency. First, global warming could create a more favorable ecosystem for *A. flavus*, as observed in the seasonal pattern in North America, which has seen increased cases in summer months (*10*). Second, migrating populations could be at risk for IA after long-term carriage of *A. flavus* previously acquired in more southern countries, as noted by the high percentage of patients originating from North Africa among our *A. flavus* IA cohort. Finally, increases in the population of patients with acquired immunodeficiency, such as organ recipients (*22*), could mean that more patients are susceptible to present *A. flavus* infection and have more severe outcomes.

In conclusion, we found that IA caused by *A. flavus* shared many similarities with IA caused by *A. fumigatus*. Clinical manifestations mainly showed 2 distinct patterns: aggressive pleuropulmonary or disseminated infection in highly immunocompromised patients, with a high rate of co-infection and a high mortality rate despite antifungal treatment; and ENT infections, usually occurring in patients with uncontrolled diabetes mellitus, frequently among persons originating from countries in North Africa, and showing low mortality rates after curative surgery and antifungal treatment. Surveillance studies such as ours can raise awareness of non–*A.* *fumigatus* IA, including in Western countries, where their incidence may increase in the future because of climate change. Clinicians should remain vigilant for *A. flavus* and consider it during differential diagnosis for IA. 
